# Leptin and adiponectin in relation to body fat percentage, waist to hip ratio and the apoB/apoA1 ratio in Asian Indian and Caucasian men and women

**DOI:** 10.1186/1743-7075-3-18

**Published:** 2006-04-10

**Authors:** Jessica Smith, Maha Al-Amri, Allan Sniderman, Katherine Cianflone

**Affiliations:** 1 Mike Rosenbloom Laboratory for Cardiovascular Research, McGill University Health Centre, Montreal, Canada; 2 Centre de Recherche Hôpital Laval, Ste-Foy, Québec, Canada

## Abstract

**Background:**

Asian Indian immigrants have an increased risk for developing cardiovascular disease (CVD); however, there is very little data examining how the adipokines leptin and adiponectin relate to CVD risk factors such as body fat percentage (BF%), waist to hip ratio (WHR) and the apoB/apoA1 ratio in Asian Indian men and women living in Canada.

**Subjects and methods:**

A cross-sectional study comparing leptin, adiponectin, lipoproteins and anthropometric parameters in Asian Indian men and women to Caucasian men and women (4 groups). Anthropometric data (BMI, BF%, WHR), circulating lipids (apoA1, apoB, total cholesterol, and HDL-cholesterol), leptin and adiponectin were measured.

**Results:**

Asian Indian men and women had higher leptin and lower adiponectin concentrations then Caucasian men and women, respectively. Leptin (positively) and adiponectin (negatively) correlated with anthropometric parameters and lipoproteins in all four groups. Using stepwise forward multiple regression, a model including TC/HDL-C ratio, WHR, BF%, hip circumference and waist circumference predicted 74.2% of leptin concentration in men. In women, apoB, BF%, waist circumference and age predicted 77.5% of leptin concentration. Adiponectin concentrations in men were predicted (30.2%) by HDL-C, total cholesterol, hip circumference and BF% while in women 41.2% of adiponectin concentration was predicted by the apoB/apoA1 ratio, WHR and age.

**Conclusion:**

As is evident from our data, there is a strong relationship between leptin, adiponectin, and abdominal obesity with increased CVD risk, as assessed by the apoB/apoA1 ratio. Dysregulation of these parameters may account for the increased risk of CVD in Asian Indians.

## Background

Asian Indian men and women have a higher incidence and mortality rate from cardiovascular disease (CVD) than Caucasian men and women, yet the pathology behind this increased susceptibility is not fully understood [1-3]. Within India there has been a drastic increase in the incidence of CVD as a result of improving social and economic conditions [4,5]. Even immigrants within the same environment, when compared to Europeans or Caucasians, have a 1.5 to 4.0 times higher mortality rate from CVD and this risk increases with duration of residence [2,6,7].

Factors such as body fat percentage (BF%), body fat distribution, dyslipidemia, and adipose tissue derived hormones (specifically, leptin and adiponectin) all impact the development of atherogenesis and CVD [8-12]. It has been established that Asian Indians have a higher BF% for the same BMI when compared to Caucasians [13,14]. Lower BMI cut-points have been suggested to evaluate overweight and obesity specifically for Asian populations [15] since this increased body fat at lower BMI has been associated with negative metabolic consequences [16].

Excess adiposity has variable effects from individual to individual. Why some individuals or groups, such as Asian Indians, have a greater propensity for dyslipidemia with weight gain and why some people with obesity avoid the common metabolic complications of dyslipidemia and insulin resistance is only beginning to be understood. Adipose tissue distribution and adipose tissue derived hormones may play an important role in distinguishing those who develop complications from those who do not.

Abdominal fat has several characteristics that distinguish it from lower body fat and it is an important modulator of the relationship between BF% and CVD [8,10,17]. Several correlative and predictive studies have shown that increased abdominal fat increases one's risk for several complications of obesity including insulin resistance, metabolic syndrome, type 2 diabetes mellitus and ultimately, CVD [18-20].

In addition to body composition and lipid profile, various adipose tissue hormones have recently been shown to impact CVD risk. Leptin and adiponectin are influenced by body fat status, with associations to CVD [11,12,21]. Leptin has been shown to be a predictor of CVD in both case-control and prospective studies [11,21] and adiponectin negatively correlates with plasma triglycerides (TG) and positively correlates with HDL-C [12]. Yet there is limited data in Indians; therefore, the aim of the present study is to evaluate how the adipokines leptin and adiponectin related to body composition and CVD risk in Asian Indians.

## Subjects and methods

Men and women living in Montreal, Canada between the ages of 20 to 60 were recruited for participation in this study. Subjects were excluded if they had a history of CVD or were taking lipid lowering medication. Subjects identified themselves as Asian Northern Indian or Caucasian.

Caucasian subjects were recruited through advertisements posted in McGill University Heath Centre (Montreal QC) and in local newspapers. Northern Indian subjects were recruited through local community centers. Ethics approval for this project was obtained from the McGill University Heath Centre (Royal Victoria Hospital) ethics review committee (Montreal QC) and subjects signed an informed consent form prior to participation.

### Study design

The study design was cross-sectional. Data was collected between November 2004 and March 2005. Anthropometric parameters and blood samples were collected at a single visit at the McGill University Heath Centre (MUHC, Royal Victoria Hospital), Montreal QC or in community centers in Montreal. Demographic information was collected using a self-administered questionnaire.

### Anthropometric measurements

Height and weight were measured to the nearest 0.1 kg and 0.5 cm, respectively. Waist circumference was measured at the horizontal circumference between the lowest rib margin and the iliac crest and hip circumference was measured at the maximum circumference over the buttocks. Body mass index (BMI) was calculated as weight (kg) divided by height (m) squared. Waist to hip ratio (WHR) was calculated as waist circumference divided by hip circumference.

A tetrapolar bioelectrical impedance device (RJL Systems, Clinton Townships, MI) was used to measure body composition. Resistance and reactance measurements were recorded from the device and body fat mass (kg) and BF% were calculated using manufacturer's software according to their protocols. Measurements were taken with the subjects sitting. Hydration status can influence the results, therefore, subjects were asked to refrain from intense exercise or excessive alcohol consumption for 3 days prior to the assessment.

### Blood lipids and adipokines

Venous blood samples (10 mL) were collected in the non-fasting state into non-heparinized and EDTA free tubes. Blood samples were centrifuged at 2000 rpm at 4°C for 10 minutes. Serum to be analyzed for total cholesterol (TC), high density lipoprotein cholesterol (HDL-C), apolipoprotein B (apoB) and apolipoprotein A1 (apoA1) was stored for less than 12 hours at 4°C. The central laboratory at the Royal Victoria Hospital (Montreal QC) conducted all assays for apolipoproteins and blood lipids using standard laboratory methods. The normal range for each parameter is as follows: TC, 3.6 to 6.2 mmol/L; HDL-C, 0.9 to 2.4 mmol/L; apoB, 0.50 to 1.40 g/L; and apoA1, 0.90 to 1.80 g/L. A second aliquot of serum was stored at -80°C for analysis of adipokines. Adiponectin and leptin were measured using radioimmunoassay (RIA) and, based on our data, the CVs were 6.2% and 8.3% respectively (Linco, St. Charles, MI).

### Statistical analysis

All results were expressed as mean ± standard error of the mean (SEM). All statistical analysis was done using SigmaStat (Jandel, San Rafael, CA) and GraphPad Prism (San Diego CA). Continuous data were compared for Indian men vs. Caucasian men or Indian women vs. Caucasian women for each parameter by unpaired two-tailed t-test. For proportional data, Fisher exact test was used to provide a more conservative estimate of significance. Relationships between variables in each group were assessed by linear regression analysis using Pearson correlation and predictive models were assessed using stepwise forward multiple regression analysis. Significance was set at p < 0.05.

## Results

Table 1 shows the anthropometric, lipid and lifestyle characteristics of the Indian and Caucasian men and women. There was no significant difference in the age of the women; however, the Indian men were significantly older than the Caucasian men.

**Table 1:** Lifestyle, Anthropometric and Blood Lipid Characteristics. Values are reported as mean ± standard error of the mean (SEM). P-values are reported for Fisher exact test (†) or unpaired t-test (*) between Indian men vs Caucasian men or Indian women vs Caucasian women for each parameter with a p-value of less than 0.05 considered significant

	**Indian Men**	**Caucasian Men**	**P**	**Indian Women**	**Caucasian Women**	**P**
**N**	54	32		28	51	
**Born in Canada (yes/no) %**	0/52 0%	29/3 90.6%	< 0.001†	1/27 3.6%	38/13 74.5%	< 0.001†
**Diabetes (yes/no) %**	8/42 16.0%	1/31 3.1%	NS†	0/27 0%	0/51 0%	NS†
**Smoking (yes/no) %**	4/46 8.0%	9/23 28.1%	NS†	1/26 3.7%	25/25 50.0%	0.01†
**Age (years)**	42.9 ± 1.34	38.3 ± 1.73	0.04*	43.0 ± 1.95	40.3 ± 1.55	NS*
**BMI (kg/m**^2^**)**	28.3 ± 0.58	26.6 ± 0.70	NS*	28.7 ± 0.86	25.8 ± 0.72	0.01*
**BF%**	21.6 ± 0.65	17.6 ± 0.89	0.0005*	40.1 ± 1.52	33.6 ± 1.23	0.002*
**WHR**	0.93 ± 0.01	0.86 ± 0.01	< 0.0001*	0.88 ± 0.01	0.77 ± 0.01	< 0.0001*
**WC (cm)**	100.4 ± 1.3	91.1 ± 2.1	0.0002*	94.4 ± 2.3	83.0 ± 2.2	0.001*
**HC (cm)**	108.3 ± 0.9	106.3 ± 1.2	NS*	107.0 ± 2.0	106.4 ± 1.6	NS*
**apoB (g/L)**	1.00 ± 0.03	0.79 ± 0.05	0.0005*	0.92 ± 0.04	0.77 ± 0.03	0.002*
**apoA1 (g/L)**	1.19 ± 0.03	1.23 ± 0.04	NS*	1.30 ± 0.05	1.44 ± 0.04	0.05*
**TC (mmol/L)**	5.23 ± 0.13	4.79 ± 0.19	NS*	5.27 ± 0.16	5.02 ± 0.13	NS*
**HDL-C (mmol/l)**	0.97 ± 0.03	1.24 ± 0.05	< 0.0001*	1.13 ± 0.05	1.51 ± 0.06	< 0.0001*
**TC/HDL-C**	5.44 ± 0.21	3.90 ± 0.23	< 0.0001*	4.64 ± 0.24	3.37 ± 0.16	< 0.0001*
**apoB/apo A1**	0.85 ± 0.03	0.66 ± 0.04	0.0002*	0.73 ± 0.04	0.56 ± 0.03	0.0003*

BMI = body mass index; BF% = body fat percentage; WHR = waist to hip ratio; WC = waist circumference; HC = hip circumference; apoB = apolipoprotein B; apoA1 = apolipoprotein A1; TC = total cholesterol; HDL-C = high density lipoprotein cholesterol; TC/HDL-C = TC to HDL-C ratio; apoB/apoA1 = apoB to apoA1 ratio.

Indian men had less current or former smokers than Caucasian men (8.0% vs. 28.1%, NS) although there was no significant difference; however, there were fewer Indian women who were current or former smokers compared to Caucasian women (3.7% vs. 50.0%, p = 0.01). None of the Indian or Caucasian women were diabetic. Of the Indian men, 16.0% were diabetic while 3.1% of Caucasian men were diabetic. None of the Indian men were born in Canada and those that had immigrated had an average of 13.9 ± 1.4 years living in Canada. Only 1 (3.6%) Indian woman was born in Canada and the remaining women had lived in Canada for an average of 11.9 ± 1.6 years. Considerably more Caucasian subjects were born in Canada with 90.6% of Caucasian men born in Canada and 74.5% of Caucasian women born in Canada.

There was no difference in BMI between Indian and Caucasian men; however, Indian women had a significantly higher (11%) BMI then the Caucasian women. Both the Indian men and Indian women had a significantly higher BF% then the Caucasian men and women, respectively. There were striking differences in the WHR between the Indian and Caucasian subjects for both women and men (men 0.93 ± 0.01 vs. 0.86 ± 0.01, p < 0.0001; women: 0.88 ± 0.01 vs. 0.77 ± 0.01, p < 0.0001).

There was no difference between either men or women for TC; however, Indian men and women had significantly higher apoB, TC/HDL-C ratio and apoB/apoA1 ratio, and significantly lower HDL-C, than Caucasian men and women. Indian women had lower apoA1 than Caucasian women but there was no significant difference between Indian and Caucasian men. Thus, the Indian men and women have a much less favourable lipoprotein profile than do Caucasians, indicating an elevated CVD risk.

**Figure 1:**
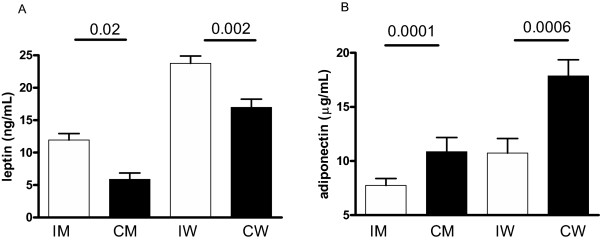
Non-fasting serum concentrations of leptin (A) and adiponectin (B) in Indian Men (IM), Caucasian men (CM), Indian women (IW) and Caucasian women (CW). Bars represent mean ± SEM. P-value calculated by two-tailed t-test for IM vs CM and IW vs CW.

Because of the differences in body fat distribution, we then examined serum adipokines. In addition to the differences seen in anthropometric profile and lipoproteins, pronounced differences were seen in adipokines (Figure 1). Leptin was significantly higher and adiponectin levels significantly lower in the Indian men compared to the Caucasian men (leptin: 11.94 ± 0.01 ng/mL vs. 5.88 ± 0.97 ng/mL, p = 0.0001; adiponectin: 7.75 ± 0.64 μg/mL vs. 10.85 ± 1.32 μg/mL, p = 0.02). This was also true in Indian women compared to the Caucasian women (leptin: 23.76 ± 1.13 ng/mL vs. 16.97 ± 1.27 ng/mL, p = 0.0006; adiponectin: 10.73 ± 1.35 μg/mL vs. 17.86 ± 1.49 μg/mL, p = 0.002).

Leptin correlated positively with all body composition parameters, including BMI, BF%, WHR, waist circumference and hip circumference, in all groups, except WHR in Indian women (Table 2). In addition, when data was pooled for all women together and for all men together, we found similar results: leptin correlated positively with body composition parameters. Interestingly, leptin also correlated with lipids and lipoproteins: TC in Caucasian women and Indian men, apoB in Indian men and apoB/apoA1 ratio in Caucasian women and Indian men. Again, we found similar correlations when data was pooled and analyzed for all men together and all women together (Table 3).

**Table 2:** Pearson correlation of leptin with body composition parameters and lipoproteins in Indian men, Caucasian men, Indian women and Caucasian women

**R P**	**Indian Men**	**Caucasian Men**	**Indian Women**	**Caucasian Women**
BMI	***0.72 < 0.0001***	***0.67 < 0.0001***	***0.54 0.003***	***0.81 < 0.0001***
BF%	***0.63 < 0.0001***	***0.62 0.0002***	***0.52 0.005***	***0.85 < 0.0001***
WHR	***0.49 0.0002***	***0.61 0.0003***	0.15 NS	***0.56 < 0.0001***
WC	***0.79 < 0.0001***	***0.68 < 0.0001***	***0.60 0.0007***	***0.78 < 0.0001***
HC	***0.71 < 0.0001***	***0.54 0.002***	***0.69 < 0.0001***	***0.82 < 0.0001***
apoB	***0.45 0.002***	0.07 NS	0.26 NS	***0.42 0.003***
apoA1	0.05 NS	***0.40 0.03***	0.26 NS	0.00 NS
apoB/apoA1	***0.41 0.005***	0.31 NS	0.01 NS	***0.31 0.03***
HDL-C	0.23 NS	-0.21 NS	0.32 NS	-0.12 NS
TC	***0.31 0.03***	0.06 NS	0.33 NS	***0.40 0.005***
TC/HDL-C	***0.44 0.001***	0.19 NS	-0.13 NS	***0.38 0.007***
Age	0.13 NS	0.21 NS	0.06 NS	0.10 NS

Bold and italicized text indicates significant values. BMI = body mass index; BF% = body fat percentage; WHR = waist to hip ratio; WC = waist circumference; HC = hip circumference; apoB = apolipoprotein B; apoA1 = apolipoprotein A1; TC = total cholesterol; HDL-C = high density lipoprotein cholesterol; TC/HDL-C = TC to HDL-C ratio; apoB/apoA1 = apoB to apoA1 ratio.

**Table 3:** Pearson correlation of adiponectin and leptin with body composition parameters and lipoproteins in men and women. Data from Indian men and Caucasian men were pooled for analysis in men and data from Indian women and Caucasian women were pooled for analysis in women

**R P**	Men: leptin	Women: leptin	Men: adiponectin	Women: adiponectin
BMI	***0.71 < 0.0001***	***0.76 < 0.0001***	-0.10 NS	***-0.23 0.04***
BF%	***0.68 < 0.0001***	***0.79 < 0.0001***	-0.18 NS	***-0.23 0.047***
WHR	***0.61 < 0.0001***	***0.59 < 0.0001***	***-0.31 0.004***	***-0.45 < 0.0001***
WC	***0.78 < 0.0001***	***0.77 < 0.0001***	-0.18 NS	***-0.34 0.003***
HC	***0.66 < 0.0001***	***0.72 < 0.0001***	0.04 NS	-0.15 NS
apoB	***0.42 0.0002***	***0.46 < 0.0001***	-0.13 NS	-0.23 NS
apoA1	-0.19 NS	-0.04 NS	***0.23 0.04***	***0.26 0.03***
apoB/apoA1	***0.48 < 0.0001***	***0.35 0.002***	***-0.25 0.03***	***-0.33 0.005***
HDL-C	***-0.36 0.001***	-0.20 NS	***0.37 0.0008***	***0.35 0.002***
TC	***0.31 0.01***	***0.40 0.0004***	0.00 NS	-0.10 NS
TC/HDL-C	***0.50 < 0.0001***	***0.36 0.001***	***-0.23 0.04***	***-0.37 0.0009***
Age	***0.24 0.03***	0.13 NS	-0.03 NS	0.19 NS

Bold and italicized text indicates significant values. BMI = body mass index; BF% = body fat percentage; WHR = waist to hip ratio; WC = waist circumference; HC = hip circumference; apoB = apolipoprotein B; apoA1 = apolipoprotein A1; TC = total cholesterol; HDL-C = high density lipoprotein cholesterol; TC/HDL-C = TC to HDL-C ratio; apoB/apoA1 = apoB to apoA1 ratio.

Adiponectin correlated with several body composition parameters (Table 4). WHR correlated negatively with adiponectin in Indian men and in a pooled analysis with data from both Indian and Caucasian men. Unexpectedly, adiponectin positively correlated with BF% and BMI in Indian women; however, in Caucasian women, adiponectin positively correlated with hip circumference and negatively correlated with waist circumference, WHR, BF%, and BMI, which is more consistent with published data. When data was pooled from Caucasian and Indian women, adiponectin correlated negatively with waist circumference, WHR, BMI and BF% (Table 3).

**Table 4:** Pearson correlation of adiponectin with body composition parameters and lipoproteins in Indian men, Caucasian men, Indian women and Caucasian women

**R P**	Indian Men	Caucasian Men	Indian Women	Caucasian Women
BMI	-0.02 NS	-0.08 NS	***0.41 0.03***	***-0.33 0.02***
BF%	-0.11 NS	-0.07 NS	***0.43 0.02***	***-0.31 0.03***
WHR	***-0.34 0.01***	-0.16 NS	-0.11 NS	***-0.33 0.02***
WC	-0.14 NS	-0.08 NS	0.19 NS	***-0.37 0.01***
HC	0.12 NS	0.04 NS	0.30 NS	***-0.33 0.02***
ApoB	-0.14 NS	0.05 NS	0.17 NS	-0.23 NS
apoA1	0.16 NS	0.27 NS	***0.64 0.0006***	0.08 NS
apoB/apoA1	-0.23 NS	-0.11 NS	-0.32 NS	-0.20 NS
HDL-C	0.10 NS	***0.41 0.02***	***0.53 0.004***	0.16 NS
TC	-0.08 NS	0.21 NS	0.26 NS	-0.16 NS
TC/HDL-C	-0.17 NS	-0.18 NS	-0.38 NS	-0.27 NS
Age	-0.03 NS	0.09 NS	0.39 0.04	0.20 NS

Bold and italicized text indicates significant values. BMI = body mass index; BF% = body fat percentage; WHR = waist to hip ratio; WC = waist circumference; HC = hip circumference; apoB = apolipoprotein B; apoA1 = apolipoprotein A1; TC = total cholesterol; HDL-C = high density lipoprotein cholesterol; TC/HDL-C = TC to HDL-C ratio; apoB/apoA1 = apoB to apoA1 ratio. Bold and italicized text indicates significant values. BMI = body mass index; BF% = body fat percentage; WHR = waist to hip ratio; WC = waist circumference; HC = hip circumference; apoB = apolipoprotein B; apoA1 = apolipoprotein A1; TC = total cholesterol; HDL-C = high density lipoprotein cholesterol; TC/HDL-C = TC to HDL-C ratio; apoB/apoA1 = apoB to apoA1 ratio.

Several of the lipoprotein parameters also correlated with adiponectin such as apoA1 and HDL-C in Indian women and HDL-C in Caucasian men (Table 4). When the data from Indian and Caucasian men were pooled, we found that apoA1, the apoB/apoA1 ratio, HDL-C, and the TC/HDL-C ratio, correlated with adiponectin. Likewise, in a pooled analysis of women, apoB, apoA1, the apoB/apoA1 ratio, HDL-C, and the TC/HDL-C ratio correlated with adiponectin (Table 3).

We next examined which parameters significantly predict leptin and adiponectin concentrations, using stepwise forward multiple regression analysis. In a pooled analysis of Indian men and Caucasian men (to provide a wide range of effects), a model including HDL-C, total cholesterol, BF%, WHR, hip circumference and waist circumference predicted 74.2% of leptin concentration. In women a combination of apoB, BF%, waist circumference and age were the strongest model, predicting 77.5% of leptin concentrations.

Adiponectin concentration in men was predicted by a model including HDL-C, total cholesterol, BF% and hip circumference (30.2%) while in women apoB/apoA1, WHR and age predicted 41.2% of adiponectin concentrations. In all cases, each parameter included contributed independently and significantly to the model.

## Discussion

Our results indicate that Asian Indian men and women have a high CVD risk profile based on both anthropometric parameters and lipid profile (higher apoB/apoA1 ratio, BF% and WHR). These two components are further contributed to by abnormal adipokine levels (higher leptin and lower adiponectin) relative to a comparable Caucasian group. Despite the close relationship between CVD, body composition and the adipokines leptin and adiponectin, there is very little data in Asian Indian immigrants on how these factors interrelate.

In Caucasian populations, both leptin (positively) and adiponectin (negatively) have been shown to correlate with CVD risk and BMI, BF% and WHR [11,12,21]. Increases in body fat are strongly linked to adipokine production, specifically that of leptin and adiponectin [12,22]. Increases in BF% and WHR are associated with dyslipidemia, including increased plasma TG, NEFA and apoB [23]. Adipokines may also have independent effects on CVD risk, separate from their associations with obesity; however conflicting data exists and exact mechanisms have not been elucidated [24].

There are a limited number of papers which suggest that the same patterns of association between leptin, adiponectin, and CVD risk factors may be seen in Asian Indian immigrants [16,25,26]. Several studies have been conducted in India which have shown relationships between insulin resistance, body composition, lipoproteins, leptin and adiponectin [27,28]; yet for our purposes, only papers with data from Asian Indian immigrants will be discussed. To our knowledge, the present study is the only one that examines adipokines, apoproteins and anthropometric values in both men and women with a direct comparison of Asian Indian to Caucasian. Nonetheless, these previous studies, taken together with our present data, provide some valuable insights.

We found that both Asian Indian men and women had significantly higher leptin levels and significantly lower adiponectin levels then Caucasian men and women, respectively. The only other study examining leptin and adiponectin in Asian Indian immigrants to North America found that Indian men had higher leptin and lower adiponectin, which supports our findings [25]. However, this study by Abate *et al*. only included young, relatively lean men and no women. Interestingly, in that study, in spite of a smaller waist circumference yet similar BMI to Caucasian men, Asian men still had increased leptin and decreased adiponectin [25]. A study conducted in South Africa found that Asian Indian men and women had lower adiponectin (corrected for age and WHR) than BMI matched white men and women [26]. However, it is difficult to draw specific comparisons between this study and ours because the multiple regression analysis in that study was done using pooled data from both men and women in spite of the well known gender differences in leptin and adiponectin. Also, adjusted averages are reported for many of the parameters.

We also found strong correlations between leptin and all measures of body composition (BMI, BF%) and body fat distribution (WHR, waist circumference, hip circumference) which confirms that the same associations seen with leptin and body composition are present in Asian Indian immigrants. Also, at least one measure of body fat distribution (WHR, waist circumference or hip circumference) was a significant predictor of leptin or adiponectin concentration in stepwise forward multiple regression analysis.

Abate *et al *found similar correlations between leptin and BF% and waist circumference in lean Asian Indian men [25]. An additional study, also with young Asian Indian men now living in the US, found that leptin correlated with total and subcutaneous fat, but not visceral fat, yet these men had relatively small waist circumference and WHR and were younger than our population group [16]. Ferris *et al*. reported that Asian Indian men and women had lower adiponectin (corrected for age and WHR) than BMI-matched white men and women, however, the differences were only significant between women [26]. No additional studies have been published linking leptin and body composition in immigrant Indian women. Whether leptin or adiponectin are elevated in Asian Indians prior to the onset of altered body fat distribution and elevated apoB/apoA1 remains to be answered through longitudinal studies.

We found several correlations of the adipokines with lipids and lipoproteins, especially apoB, apoA1 and the apoB/apoA1 ratio, which are strong indicators of cardiovascular risk. While similar relationships with lipids have been identified with leptin and adiponectin in Asian Indians living in India, there was no direct comparison to Caucasians. In both Indian men and a pooled analysis of Indian and Caucasian men, we found that apoB and the apoB/apoA1 ratio correlated with leptin. These results have been shown in Caucasian populations yet have not been demonstrated in an immigrant Asian Indian population. Banerji *et al*. also found no correlation between leptin and plasma lipids [16], yet they did not measure apoB or apoA1 and consequently, the apoB/apoA1 ratio, which has been shown to be a more powerful predictor of CVD risk than lipid profile alone [18].

While age did not significantly contribute to the prediction of leptin or adiponectin levels in men in a multiple regression model, the higher age of the Asian Indian men may have contributed to their more adverse CVD risk profile than Caucasian men. However, several other parameters were similar, such as BMI, diabetic status and smoking status and the clinical significance of the age difference (42.9 ± 1.34 vs. 38.3 ± 1.73) is debatable, particularly since the upper limit of age was restricted to 60 years.

What was particularly striking in our present study was the increased CVD risk profile in Indian women. To our knowledge, no other studies have evaluated leptin and adiponectin in Asian Indian women in North America. Leptin, adiponectin, lipid profile (especially apoB, apoA1 and the apoB/apoA1 ratio), WHR and BF% were substantially altered compared to Caucasian women. In fact, in many ways, the Indian women resembled more closely the profile of the Caucasian men.

## Conclusion

As is evident from our data, there is a strong relationship between leptin, adiponectin, and abdominal obesity with increased CVD risk, as assessed by the apoB/apoA1 ratio. The mechanisms behind this inter-relationship will provide insight into the heterogeneous nature of obesity and the pathogenesis of CVD. Potentially, dysregulation of these parameters may account for the increased risk of Asian Indians.

## Abbreviations

apoA1 = apolipoprotein A1

apoB = apolipoprotein B

BF% = body fat percentage

BMI = body mass index

CVD = cardiovascular disease

HC = hip circumference

HDL-C = high density lipoprotein cholesterol

TC = total cholesterol

WC = waist circumference

WHR = waist to hip ratio

## Competing interests

The author(s) declare that they have no competing interests.

## Authors' contributions

JS participated in the design of the study; the collection of data, performed the data analysis and drafted the manuscript. MA participated in the collection of data. AS conceived of the study, participated in its design and coordination and KC participated in the design of the study, its coordination and data analysis as well and helped draft the manuscript. All authors read and approved the final manuscript.
